# Neuroticism and Fear of COVID-19. The Interplay Between Boredom, Fantasy Engagement, and Perceived Control Over Time

**DOI:** 10.3389/fpsyg.2020.574393

**Published:** 2020-10-13

**Authors:** Barbara Caci, Silvana Miceli, Fabrizio Scrima, Maurizio Cardaci

**Affiliations:** ^1^Department of Psychology, Educational Science and Human Movement, University of Palermo, Palermo, Italy; ^2^Département de Psychologie, Université de Rouen, Moint Saint-Aignan, France

**Keywords:** neuroticism, COVID-19, fear of COVID-19, boredom, fantasy engagement, time, time management

## Abstract

The Italian government adopted measures to prevent the spread of coronavirus 2019 (COVID-19) infection from March 9, 2020, to May 4, 2020 and imposed a phase of social distancing and self-isolation to all adult citizens. Although justified and necessary, psychologists question the impact of this process of COVID-19 isolation on the mental health of the population. Hence, this paper investigated the relationship between neuroticism, boredom, fantasy engagement, perceived control over time, and the fear of COVID-19. Specifically, we performed a cross-sectional study aimed at testing an integrative moderated mediation model. Our model assigned the boredom to the mediation role and both the fantasy engagement and perceived control of time to the role of moderators in the relationship between neuroticism and the fear of COVID-19. A sample of 301 subjects, mainly women (68.8%), aged between 18 and 57 years (*M*_age_ = 22.12 years; *SD* = 6.29), participated in a survey conducted in the 1st-week lockdown phase 2 in Italy from May 7 to 18, 2020. Results suggested that neuroticism is crucial in coping with the COVID-19 pandemic, in line with literature showing high neurotic people having greater emotional reactivity and scarce resources to manage stress. We also found that people with high neuroticism tend to feel bored, and the relationship between neuroticism and boredom seems enhanced if one is involved in negative fantasies. Therefore, this result could also explain the positive effect between boredom and fear of COVID-19 we found in the current study. However, our data show that perceived control over time moderates the association between boredom and fear toward COVID-19. Having a high perceived control over time allows people to reduce boredom’s effect on fear of COVID-19. In conclusion, we retain that psychological treatment programs could improve the individuals’ perceived control over time to modulate anxiety toward the fear of COVID-19 and promote psychological well-being.

## Introduction

Since its first identification by the Wuhan Municipal Health Commission, China, the coronavirus 2019 (COVID-19) has become a pandemic (World Health Organization, 2020). The exceptionally high infection rate and relatively high mortality led the government’s advice of many countries for all citizens to move toward self-isolation and social distancing to reduce transmission rates, the risk of severe illness, and the impact on the acute health services. Regarding Italy, the lockdown started on 9 March 2020 with a first phase (from March 9, 2020, to May 4, 2020) imposing to only one person for family, usually an adult, to go out for buying food or medicines and taking care of the relatives with special needs. Productive activities aimed to distribute and commercialize necessities (i.e., food, journals, and medicines) were allowed. Other kinds of activities were closed or permitted only with smart working.

Similarly, schools and universities were locked, and only distance learning activities were permitted. Physical or sports activities and other kinds of recreational activities such as going to the cinema, theater, pub, or restaurants were not authorized and joining friends at home. The government launched a second phase from May 4 to 18, 2020, maintaining smart working instead, but allowing some kinds of commercials (e.g., shops for baby clothes), professionals (e.g., psychologists), or factory activities. People were also left to do individual physical activities outside the houses or recreational activities such as going to restaurants for take-away and meeting relatives, but not friends at home. However, distance learning for students continued as the only permitted educational activities. Although justified and necessary, psychiatrists, and psychologists question the impact of this process of COVID-19 isolation on the mental health of the population (e.g., Lee et al., 2020). People start developing a new fear of COVID-19, as recently described by Ahorsu et al. (2020), and changing their social habits utterly and coping with new psychological demands.

From a psychological point of view, we must consider that individual dispositions could make a difference along with other contextual variables. Although considering the importance of analyzing the entire spectrum of personality traits, in the current study, we specifically focus on neuroticism as defined by the Five Factors Model (FFM; John and Srivastava, 1999; McCrae and Costa, 1999). According to FFM, neurotic people experience unpleasant emotions, such as anger, anxiety, depression, or vulnerability (John and Srivastava, 1999; McCrae and Costa, 1999). Some experimental researchers have found neuroticism is the most significant trait that leads people to more robust conditioned fear responses (e.g., Orleans-Pobee, 2017), consistently with theory of Eysenck (1965, 1967), and also suggesting that neurotic people are more sensitive to signals of punishment (Gray, 1976, 1982). Garcia and Zoellner (2017) reported that people with high levels of neuroticism perceive higher levels of risk and show attentional biases toward ambiguous stimuli. Hence, the authors suggested that both neuroticism and lack of predictability about the likelihood of feared events increase the degree to which fear generalizes (Garcia and Zoellner, 2017). A recent meta-analysis also showed that healthy individuals with high neurotic personality traits have a significantly greater generalization of fear of safe and novel cues and contexts (Sep et al., 2019). These outcomes are coherent with prior works reporting significant associations between neuroticism and adverse emotional outcomes in stressful life experiences (Penley and Tomaka, 2002). High neurotic individuals also have a high susceptibility to psychological distress (Watts et al., 2019), inefficient coping with stress, and an inability to control urges (Ormel and Wohlfarth, 1991). They are also are prone to experiencing anxiety, anger, sadness, and disgust (McCrae, 1990; Schwebel and Suls, 1999).

Besides, the lockdown state imposed by the COVID-19 pandemic has significantly changed our rhythms of life: time always flows the same, and in these conditions, one must learn to manage one’s time without using the daily “official timing” routines. Thus, the perceived control of time, defined as the perception of the individual’s control over how time has passed (Macan et al., 1990), becomes a crucial psychological variable. Scholars suggested that the individual’s perceived control over time has a mediator effect on time management behaviors on self-reported job performance, work and life satisfaction, role ambiguity, and job-induced and somatic tensions (Macan, 1994). Other studies also highlight how perceived control over time intervenes to modulate the relationship between personality dimensions and psychological well-being. Specifically, poor perceived control over time is associated with personality dimensions such as neuroticism (Feather and Bond, 1994) and psychological stress, anxiety, or depression (Griffiths, 2003; Chang and Nguyen, 2011). As well, during the COVID-19 pandemic, individuals also live a series of “empty moments” they wish to fill with new activities such as reading, watching TV, or playing videogames, so devoting considerable time and resources to the pursuit of fantasy (e.g., daydreaming or doing multiplayer nonreality games). In all these daily activities, they could live a sort of fantasy engagement that is a “conscious and deliberate suspension of disbelief in nonreality. A person is said to be engaging in fantasy if he or she chooses to engage with an instance of nonreality as though it were reality. For example, a person watching a film portraying fictional events has been engaging in fantasy. Viewers likely know that the on-screen events are not happening. Nevertheless, they can choose to temporarily suspend this disbelief or awareness in the nonreality status of the film, allowing them to experience authentic affective responses to its content (e.g., crying, exhilaration)” (Plante et al., 2017, p. 1).

However, we must distinguish between negative and positive fantasy engagement. The former refers to fantasies with harmful contents such as violence, sex, or antisocial themes that can amplify addiction and desire (Andrade et al., 2012), leading people to maladaptive or dysfunctional behaviors. The latter refers to positive themes and has potential benefits, including healthy childhood development, motivating goal pursuit, and physical and psychological well-being (Overby, 2013). Styles of thought aimed at elaborating fantasies (i.e., the fantasy engagement) such as the daydreaming activities or the elaboration of possible scenarios, while watching a film or reading a book, can both represent useful mental distractors from perceived stress situations and to promote motivating actions in subjects aimed at implementing health promotion behaviors such as exercise, or healthy diet (Sheeran et al., 2013).

Finally, we argue that coping with COVID-19 pandemic individuals might also feel annoyed or bored. This detrimental impact of boredom may, in turn, lead them to cope with various mental health conditions, such as traumatic head injury (e.g., Seel and Kreutzer, 2003), depression and anxiety (Sommers and Vodanovich, 2000), apathy (Ahmed, 1990), negative affect (Gordon et al., 1997), hostility and anger (Rupp and Vodanovich, 1997), job dissatisfaction (Kass et al., 2001), and low achievement in school (Jarvis and Seifert, 2002).

**Figure 1:**
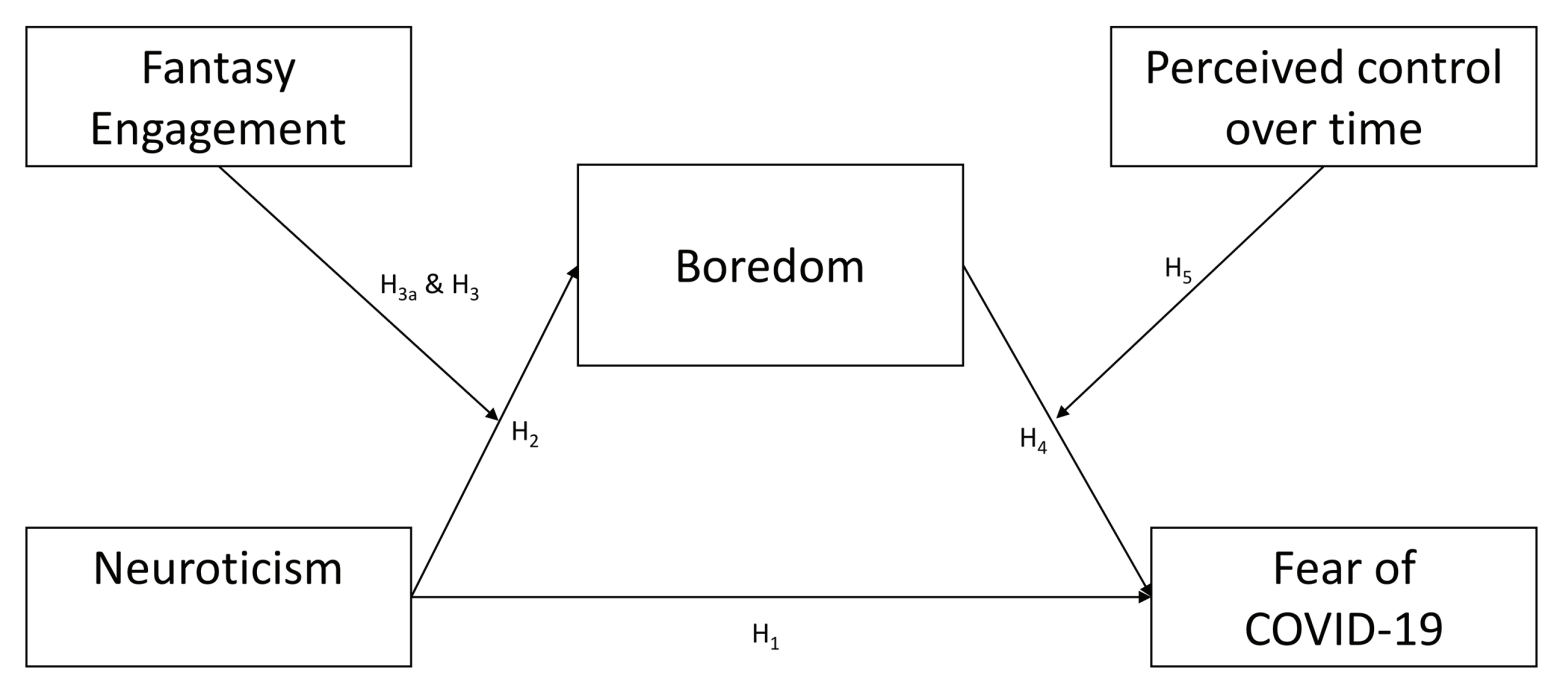
Theoretical model.

Starting from the state of the art above-described, in the current study, we aimed at analyzing the relationship between neuroticism, boredom, the fantasy engagement, the perceived control over time, and the fear of COVID-19 during the phase of social distancing. Specifically, we tested a mediation-moderation model (Figure 1), assigning the boredom to the mediation role and both the fantasy engagement and perceived control of time to the role of moderators in the relationship between neuroticism and the fear of COVID-19. Explicitly, we stated the following hypotheses:

H1: Neuroticism is positively related to fear of COVID-19.
H2: Neuroticism is positively related to boredom.
H3a: Negative fantasy engagement is a moderator between neuroticism and boredom.
H3b: Positive fantasy engagement is a moderator between neuroticism and boredom.
H4: Boredom is positively related to COVID-19.
H5: Perceived control over time moderates the relationship between boredom and fear of COVID-19.
H6: Boredom mediates the relationship between neuroticism and fear of COVID-19.

## Materials And Methods

### Participants

The Bioethics Committee of the University of Palermo has approved the current study (n. 2/2020). Participants gave written consent about the anonymity of data handling, according to the Declaration of Helsinki. A total of 301 subjects, aged between 18 and 57 years, with a mean age of 22.12 years (*SD* = 6.29), participated in the survey. Most of the participants were female (68.8%). Almost all participants came from southern Italy (85.7%). According to educational levels, almost all participants completed higher education (79.1%), while some have a degree (16.3%) or a middle school diploma (4.7%).

### Procedure

The survey was made available on the distance learning university courses of the researchers. Participants were recruited by responding voluntarily to the survey administered online *via* Google Form in the 1st week of lockdown phase 2 in Italy from May 7 to 18, 2020. This choice is justified by wanting to observe the effects of more restrictive lockdown phase 1 on the studied dimensions. The Google Form presented the study information sheet in the first section. Data were automatically collected when participants filled the Google Form online, reporting the electronic version of the assessment instrument consisting of demographic questions (i.e., gender, age, and education) and apposite measures of the studied variables.

### Materials

#### Neuroticism Scale

We used the Neuroticism subscale of the 20-item Personality Inventory (PI; Caci et al., 2014) for measuring neuroticism. In general, PI is a measure of personality traits as defined by the FFM (Costa and McCrae, 1992), and it has five subscales, consisting of four items related to one of the personality factors (i.e., Extroversion, Conscientiousness, Neuroticism, Openness, and Agreeableness). Each item scored on a five-point Likert scale with anchors from 1 = strongly disagree to 5 = strongly agree. For the present study, we analyzed only data from the Neuroticism subscale (example of item: I am relatively stable from an emotional point of view). We computed the total score by averaging participants’ scores for each of the items of the scale. In the present study, a standardized Cronbach α coefficient was 0.70, similarly to those reported by Caci et al. (2014) in the first validation study.

#### Multidimensional State Boredom Scale

The Multidimensional State Boredom Scale (MSBS; Fahlman et al., 2013) is a 26-item scale measuring state boredom (example of item: I am stuck in a situation that I feel is irrelevant). It consists of five subscales (i.e., Disengagement, High Arousal, Low Arousal, Inattention, and Time Perception) with item scoring on a five-point Likert scale having anchors from 1 = strongly disagree to 5 = strongly agree. However, we assessed a general state of boredom by adding the scores given in all items. In the present study, a standardized Cronbach α coefficient of Overall boredom was 0.95.

#### Fear of COVID-19

The Fear of COVID-19 (FCV-19S) is a recent seven-item scale developed by Ahorsu et al. (2020) to measure the fear of COVID-19 in the adult population (example of item: “I am most afraid of coronavirus-19”). Each item scores on a five-point Likert scale having anchors from 1 = strongly disagree to 5 = strongly agree. We computed the total score by averaging the participants’ scores for each of the items of the scale (Cronbach α = 0.86).

#### Fantasy Engagement Scale

The Fantasy Engagement Scale (FES; Plante et al., 2017) is an eight-item scale measuring positive (PFE) and negative (NFE) facets of fantasy engagement. For instance, “Fantasizing about this makes me more creative” is a PFE item, whereas “My interest in this fantasy has caused problems with my family and me” is NFE one. Participants rated their agreement with each of the eight items on a five-point Likert scale with anchors from 1 = strongly disagree to 5 = strongly agree. We computed the total score for PFE and NFE by averaging participants’ scores obtained for each of the items of the scale. In the present study, standardized Cronbach α coefficients were 0.86 for PFE and 0.73 for NFE.

#### Perceived Control Over Time

The subscale of the Time Management Behavior Scale of Macan (1990) measures the individuals’ perception of control over their time usage (examples of item: I feel in control of my time; I must spend much time on unimportant tasks). It consists of five items scored on a five-point Likert scale with anchors from 1 = strongly disagree to 5 = strongly agree. We computed a total score by averaging the scores obtained by the participants for each of the items of the scale (Cronbach α = 0.70).

#### Data Analysis

The first step was to calculate descriptive statistics and zero-order correlations. Second, we carried out preliminary analyses to verify the moderating effects of positive and negative fantasy engagement on the relationship between neuroticism and boredom using PROCESS model 2 (Hayes, 2012). An integrative dual-stage moderated mediation model using PROCESS model 21 (Hayes, 2012) tested the research hypotheses. A dual-stage moderated mediation model is an integrative model consisting of the main effect, and one or more mediation or moderation effects. In the present study, the model tested the main effect of neuroticism on fear of COVID-19; the mediation effect in which boredom mediates the relationship between neuroticism and fear of COVID-19 (Stage 1); the moderation effects in which negative fantasy engagement moderates the effect of neuroticism on boredom and perceived control over time moderates the effect of boredom on fear of COVID-19 (Stage 2). All effects are measured simultaneously. Before testing the model, all variables were standardized (Hayes, 2013). The parameters were estimated using the bootstrap method with 1,000 samples and using a 95% confidence interval (CI) and using the percentile method bias-corrected (Hayes, 2013). The parameters are significant if the CI does not include zero. To conclude, two simple slope analyses were performed to interpret the moderation effects.

## Results

### Descriptive Statistics and Preliminary Analysis

Table 1 shows the means, standard deviations, and bivariate correlations between variables under study. The mean scores obtained are in line with the literature, except for fear of COVID-19, which is rather low (*M* = 1.77; *SD* = 0.61). This result would indicate that our sample has a little fear of the impact of COVID-19 on the most important aspects of everyday life. As for the demographic variables, only sex variable correlates with neuroticism (*r* = −0.134, *p* < 0.05) and fear of COVID-19 (*r* = −0.269, *p* < 0.01). This result evidences that females stated higher scores on the scale measuring neuroticism and had a higher fear of COVID-19 than males. Neuroticism is positively correlated (*p* < 0.001) with all the model variables except with perceived control over time with which it shows a negative relationship. If on the one hand positive fantasy engagement correlated with boredom only (*r* = 0.131, *p* < 0.05), negative fantasy engagement is positively correlated with neuroticism (*r* = 0.408, *p* < 0.01), boredom (*r* = 0.131, *p* < 0.05), and fear of COVID-19 (*r* = 0.144, *p* < 0.05) and negatively with perceived control over time (*r* = −0.353, *p* < 0.01). As PROCESS does not allow to test a model that foresees two simultaneous moderating variables on the relationship between the independent variable and the mediating variable and a moderating variable on the relationship between the mediation variable and the dependent variable, we carried out a preliminary analysis to verify whether positive and negative fantasy engagement moderates the relationship between neuroticism and boredom. The results of the moderation model (PROCESS-Model 2) suggest that positive fantasy engagement does not have a significant moderation effect [*b* = 0.014, *p* = n.s., CI: lower level (LL) = −0.078 upper level (UL) = 0.100], vice versa the negative fantasy engagement shows a significant effect (*b* = −0.104, *p* = 0.05, CI: LL = −0.192 UL = −0.018). This result, therefore, prevented us from testing Hypothesis 3b in the final model.

**Table 1:** Descriptive statistics and zero-order correlations.

		Mean	SD	1	2	3	4	5	6	7	8
1	Sex (0 = F, 1 = M)	-	-	1							
2	Age	22.120	6.293	0.039	1						
3	Education	-	-	−0.015	0.239^**^	1					
4	Neuroticism	2.605	0.730	−0.134^*^	−0.046	0.008	1				
5	Positive fantasy engagement	3.135	1.000	−0.044	−0.072	−0.020	0.027	1			
6	Negative fantasy engagement	1.741	0.739	0.008	0.046	0.079	0.408^**^	0.171^**^	1		
7	Boredom	2.641	0.854	−0.075	−0.092	0.030	0.593^**^	0.131^*^	0.394^**^	1	
8	Perceived control over time	3.323	0.709	−0.129^*^	0.034	−0.049	−0.375^**^	−0.101	−0.353^**^	−0.561^**^	1
9	Fear of COVID-19	1.769	0.692	−0.269^**^	−0.033	−0.024	0.255^**^	0.008	0.144^*^	0.266^**^	−0.127^**^

*N* = 301;

* *p* < 0.05;

** *p* < 0.01.

### Hypothesis Tests

**Figure 2:**
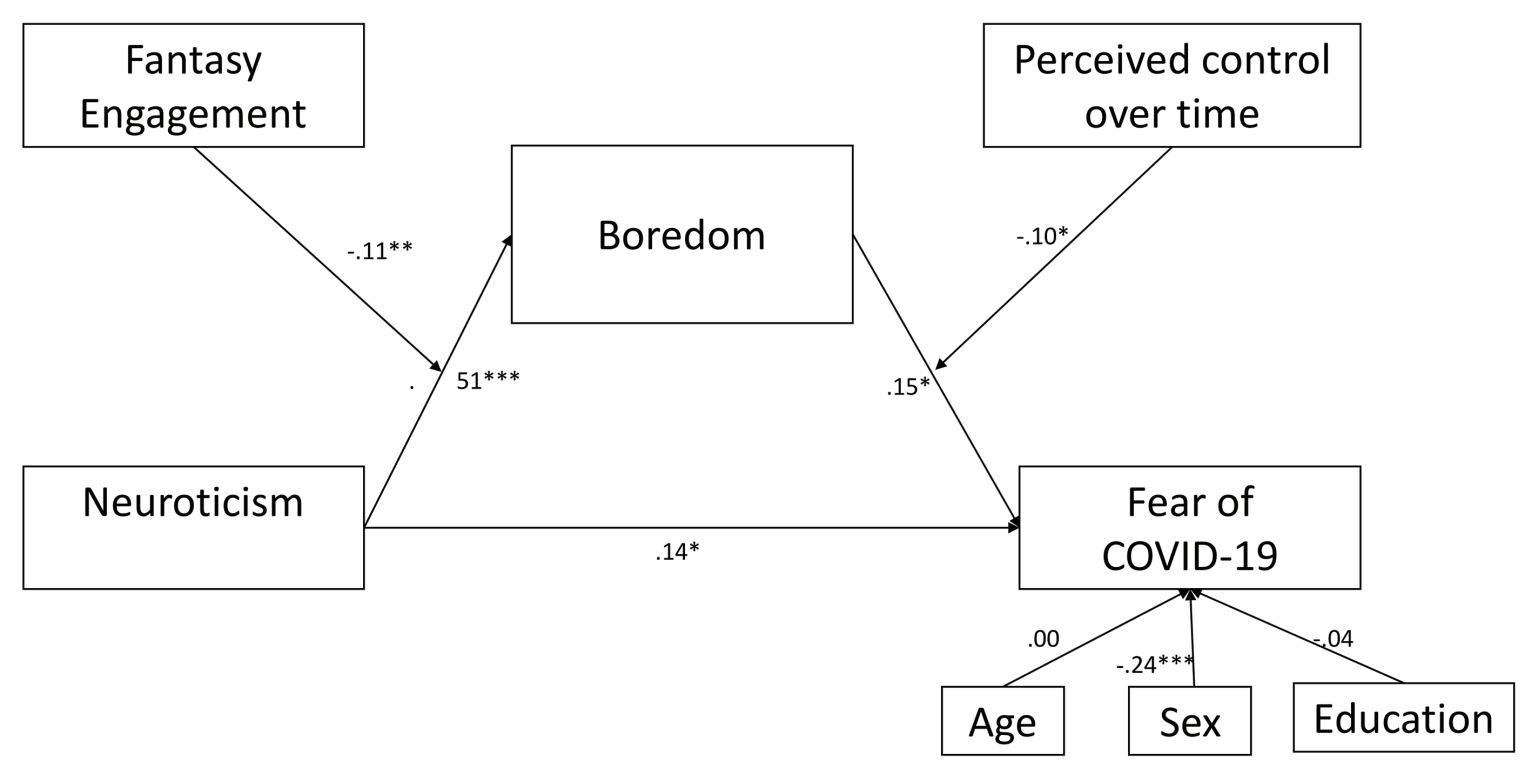
Empirical model.

Table 2 shows the results in two steps of the research hypotheses. In Step 1, regressions results are reported without the interaction terms; in Step 2, the interaction terms have been added. An *F* test was used on the variation of *R*^2^ between Step 1 and Step 2 to verify if the interaction terms bring a greater understanding of the phenomenon. Hypothesis 1 predicted that neuroticism is significantly associated with fear of COVID-19. As shown in Figure 2 and Table 2, neuroticism is positively associated with fear of COVID-19 (*b* = 0.136, *p* < 0.05, CI: LL = 0.004 UL = 0.268). This result would mean that subjects with higher neuroticism scores tend to be more feared about the impact of COVID-19 on daily life. Hypothesis 2 predicted that neuroticism is positively associated with boredom. This hypothesis is also confirmed (*b* = 0.512, *p* < 0.001, CI: LL = 0.415 UL = 0.613). The more neurotic subjects would tend to feel more bored. Hypothesis 3a established that negative fantasy engagement moderates the direct relationship between neuroticism and boredom. This hypothesis is confirmed, in fact, it has a negative and significant interaction effect (*b* = −0.115, *p* < 0.01, CI: LL = −0.199 UL = −0.029).

**Table 2:** Coefficient estimates for the moderated mediation model.

	Dependent variable: boredom						Dependent variable: fear of COVID-19					
	Step 1			Step 2			Step 1			Step 2		
	*B*	SE	*t*	*B*	SE	*t*	*B*	SE	*t*	*B*	SE	*t*
*Constant*	0.003	0.057	0.000	0.047	0.048	0.962	0.002	0.056	0.000	−0.054	0.060	−0.892
Neuroticism	0.512	0.051	10.104^***^	0.514	0.050	10.247^***^	0.114	0.068	1.682	0.136	0.067	2.032^*^
Negative fantasy engagement	0.186	0.050	3.702^***^	0.245	0.055	4.496^***^						
NEU × NFE				−0.115	0.043	−2.651^**^						
Boredom							0.170	0.076	2.238^*^	0.151	0.076	1.992^*^
Perceived control over time							−0.022	0.067	−0.335	−0.016	0.067	−0.247
BOR × PCOT										−0.100	0.049	−1.982^*^
Covariates												
Sex	−0.070	0.058	−1.218	−0.002	0.046	−0.048	−0.245	0.056	−4.388^***^	−0.240	0.055	−4.297^***^
Age	−0.102	0.059	−1.726	−0.083	0.047	−1.780	0.007	0.056	0.129	0.004	0.057	0.085
Educational level	0.054	0.059	0.905	0.024	0.047	0.505	−0.036	0.056	−0.654	−0.041	0.055	−0.735
*R*^2^	0.386			0.400			0.144			0.155		
*R*^2^ change				0.014						0.011		
*F*				7.027						3.942		
*p*				<0.01						<0.05		

*N* = 301;

* *p* < 0.05;

** *p* < 0.01;

*** *p* < 0.001.

**Figure 3:**
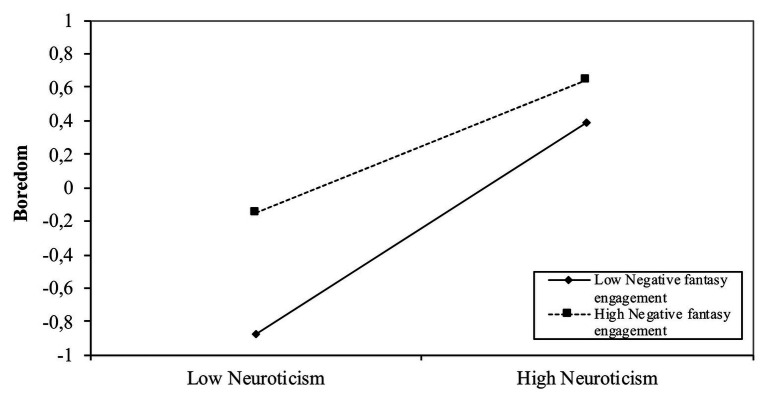
The effect of interaction between neuroticism and negative fantasy engagement on boredom.

The simple slope analysis (Figure 3) indicates that subjects with high neuroticism and more engaged in negative fantasies are those who will be most bored; conversely, subjects with low levels of neuroticism and low tendency to implicate themselves in negative fantasies tend to have shallow levels of boredom.

Hypothesis 4 stated that boredom has a direct effect on fear of COVID-19. This hypothesis is confirmed. Boredom is positively associated with fear of COVID-19 (*b* = 0.15, *p* < 0.05, CI: LL = 0.002 UL = 0.301).

**Figure 4:**
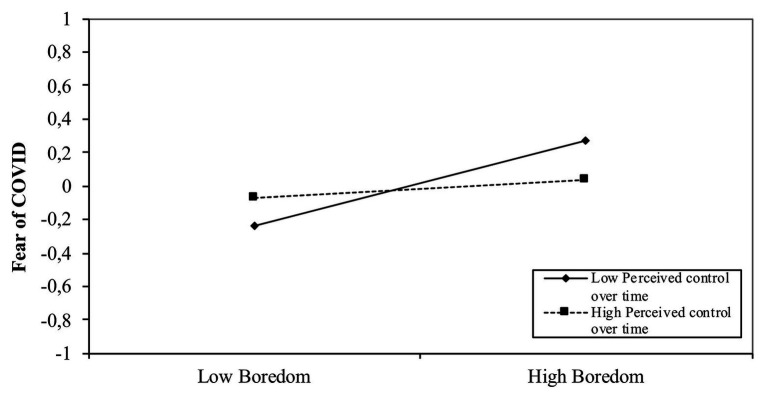
The effect of interaction between boredom and perceived control over time on fear of coronavirus 2019 (COVID-19).

Hypothesis 5 established the effect of the moderating role of perceived time control on the relationship between boredom and fear of COVID-19. In fact, a negative significant effect is indicated in Table 2 (*b* = −0.10, *p* < 0.05, CI: LL = −0.193 UL = −0.001). As shown in Figure 4, subjects with high boredom and low perceived control over time tend to have higher scores on the fear of the COVID-19 scale than subjects with low boredom and low perceived control over time.

Hypothesis 6 corroborated the entire model’s indirect conditional effects (Table 3). This analysis indicates three stages where significant effects are present. These stages coincide with low scores on the perceived control over time scale, confirming the moderated mediation effect of the entire model.

**Table 3:** Bootstrap results for the conditional indirect effects.

Negative fantasy engagement	Perceived control over time	Effect	SE	LLCI	ULCI
Low	Low	0.157^*^	0.073	0.023	0.309
Low	Mid	0.089	0.055	−0.018	0.201
Low	High	0.038	0.061	−0.087	0.160
Mid	Low	0.138^*^	0.063	020	0.268
Mid	Mid	0.078	0.048	−0.015	0.174
Mid	High	0.033	0.054	−0.076	0.139
High	Low	0.099^*^	0.047	0.013	0.196
High	Mid	0.056	0.035	−0.010	0.129
High	High	0.024	0.040	−0.054	0.103

* Significant conditional indirect effect.

## Discussion

The current study tested a moderate-mediation model seeking suggestions to reduce perceived fear of COVID-19, given the numerous pieces of evidence of the indirect impact of COVID-19 on the mental health of worldwide populations (Li et al., 2020). We collected data in a sample of Italian participants after phase 1 of the quarantine imposed on them by the government to better cope with the virus’s spread. This lockdown led to a drastic change in lifestyle since people not only reduced at minimum their physical and social relationships but also have had to face a series of potential physical or mental health problems like distress (Cheval et al., 2020; Satici et al., 2020) as far as even suicide (Mamun and Griffiths, 2020).

Our results evidenced that neuroticism is crucial in coping with the COVID-19 pandemic. Indeed, we found that neuroticism is positively associated with the fear of COVID-19, in line with literature showing its associations with many other fears as, for example, the fear of pain (Goubert et al., 2004), the fear of loss (Blackwell et al., 2017), and the fear of death (Loo, 1984). This result confirms the descriptive definition of the neurotic personality. High neurotic people show greater emotional reactivity and have scarce resources to manage stress (Larsen and Ketelaar, 1999). Hence, in the presence of a condition where it is impossible to control the situation, likewise the COVID-19 pandemic, they will tend to develop fear (Gunthert et al., 1999).

Following the results of our second hypothesis, we also found that neuroticism is related to boredom. This result is consistent with other studies in individual differences (Mercer-Lynn et al., 2013) and could depend on the lack of purpose typical of the neurotic trait. People with high neuroticism tend to have no purpose in life, which would cause them to feel bored (Bond and Feather, 1988). Furthermore, our results show that the relationship between neuroticism and boredom seems enhanced if one is involved in negative fantasies. Plante et al. (2017) have reported a significant relationship between involvement in negative fantasies and denial of daily problems. So, the lack of purpose typical of subjects with an important neurotic trait and the tendency to deny daily problems would enhance their boredom feeling.

We also found a significant positive association between boredom and fear of COVID-19. This is probably due to the fact that boredom is a temporary state linked to a lack of external stimulation and not only an effect of individual differences (Vodanovich, 2003). Indeed, contextual factors such as monotony, repetitiveness, lack of novelty, or having little to do might cause boredom (e.g., van Hooff and van Hooft, 2017). Although boredom may sometimes instigate positive behaviors such as reflection, creativity, and prosocial behavior (van Tilburg and Igou, 2017), it is more commonly associated with individuals’ adverse outcomes. For instance, negative outcomes of boredom might include reduced motivation and effort (Pekrun et al., 2010), frustration (van Tilburg and Igou, 2017), and distress (Melamed et al., 1995; van Hooff and van Hooft, 2017). Previous works have already shown the relationship between boredom and fear (Brotherton and Eser, 2015) and reported that high feeling of boredom is related to the tendency to paranoid ideas (Von Gemmingen et al., 2003). This could, therefore, explain the positive effect between boredom and fear of COVID-19 we found in the current study.

Besides, our data show that perceived control over time moderates the association between boredom and fear toward COVID-19. Having a high perceived control over time allows people to reduce the effect of boredom on fear of COVID-19. This finding is consistent with prior works demonstrating that boredom might determine individual differences in the subjective perception of the passage of time (Watt, 1991). Specifically, high boredom individuals usually have a subjective perception of the slow passage of their “mental” time, but not a slow perception of the objective passage of the “official time” measured by the clock (Watt, 1991; Cardaci, 2000), so experiencing negative feelings or emotions. Conversely, the improvement in perceived control over time has a significant effect on modulating anxiety disorders (Gallagher et al., 2013). Moreover, it could also promote psychological well-being (Chang and Nguyen, 2011).

We must evidence that our sample’s gender composition, with a high predominance of females, might be responsible for the present results. However, our findings are in line with previous literature, which evidenced well-documented gender differences in neuroticism and showed females reporting higher scores than males (see for a review Schmitt et al., 2017). Moreover, recent study findings indicate boredom has been increasing among young over the past several years, with more significant increases among females. Such increases in the perceived levels of boredom in females are concomitant with recent increases in mental health difficulties (Weybright et al., 2020). Researchers typically find females reporting lower levels of subjective well-being and higher tendency on depression than males, mainly due to women’s enhanced negative emotional responsivity (Schmitt et al., 2017). Finally, women also have higher time management skills in different behavioral domains such as domestic outsourcing or housework shares (Craig and Baxter, 2016), and academic performance (Trueman and Hartley, 1996).

## Limitations and Future Direction

Although our tested model offers a first view for understanding the processes underlying the fear of COVID-19, the present study has some limitations to report. First, the sample consists of university students with predominantly female gender. This limit does not allow generalizing the results to the entire population. Future research is necessary on more representative samples. A second limitation is the study’s cross-sectional design, which does not determine the cause–effect relationship between the variables. A third limitation is that we have used self-report measures in the current work, causing common-method bias (Podsakoff et al., 2003). To overcome these limitations, we would carry out future studies based on a longitudinal design since they give more information about the causality of the effects and minimizes the common-method bias.

## Conclusion

Despite its intrinsic limitations, we deem that the current study results could contribute to understanding psychological variables crucial for evidencing individual differences in coping with the fear of COVID-19, helping mental health practitioners develop treatment programs in the forthcoming months. Because of the moderating role of fantasy engagement on the relationship between neuroticism and boredom, we believe that specific clinical and/or educational programs should aim to improve people’s abilities to develop positive fantasies about their future. Cultivating positive fantasies about goal completion could be an excellent motivational exercise for persisting in future goal pursuit (Oettingen and Mayer, 2002), so contrasting the negative effect of the association between neuroticism personality trait and boredom. Indeed, scholars reported motivational benefits of positive fantasies in the context of academic performance (Gollwitzer et al., 2011), exercise and healthy eating (Sheeran et al., 2013), and persistence despite adversity (Kappes et al., 2012). As well, in light of the moderating role of perceived time control on the relationship between boredom and fear of COVID-19, treatment programs in mental health should aim to improve time management strategies in individuals so balancing the negative effect of the association mentioned above. Time management behaviors positively predict psychological well-being (Macan, 1996; Griffiths, 2003; see McKee-Ryan et al., 2005).

In addition to providing essential elements for reflection about mental health programs for individuals, our results suggest practical implications for the development of social policy interventions to address situations of psychological vulnerability that, while not at the heart of the health emergency, risk producing long-term effects and high social costs. Moreover, the pandemic’s current scenario does not entirely exclude the possibility of new lockdown situations capable of significantly affecting the mental balance of young people. Hence it would be useful to develop, especially within the school context of all levels, albeit remotely, social-psychological programs that emphasize positive fantasies, creativity, time management, and motivation. Above all, young people, deprived in many cases of the necessary social face to face interactions, need to experience boredom constructively, through adequate time management. In this vein, the Italian Ministry of Education has recently published guidelines for school managers stressing the necessity of having psychologists inside the schools.

## Data Availability Statement

The raw data supporting the conclusions of this article will be made available by the authors, without undue reservation.

## Ethics Statement

The studies involving human participants were reviewed and approved by The Bioethics Committee of the University of Palermo (n. 2/2020). The patients/participants provided their written informed consent to participate in this study.

## Author Contributions

BC, FS, SM, and MC contributed to the conception and design of the study. BC, SM, and MC have carried out the data collection. FS analyzed the data, prepared figures, and tables. BC, FS, SM, and MC authored and reviewed drafts of the papers and approved the final draft. All authors contributed to the article and approved the submitted version.

### Conflict of Interest

The authors declare that the research was conducted in the absence of any commercial or financial relationships that could be construed as a potential conflict of interest.
